# Comparison of Open Repair vs. the One-Stage Hybrid Extra-Anatomic Technique for Distal Aortic Arch Disease Treatment: Mid-term Outcomes With a Risk-Adjusted Analysis

**DOI:** 10.3389/fcvm.2021.725902

**Published:** 2021-08-24

**Authors:** Zhiyu Qiao, Suwei Chen, Rutao Guo, Yongliang Zhong, Yipeng Ge, Chengnan Li, Yongmin Liu, Junming Zhu, Lizhong Sun

**Affiliations:** ^1^Department of Cardiovascular Surgery, Beijing Aortic Disease Center, Beijing Anzhen Hospital, Capital Medical University, Beijing, China; ^2^Beijing Institute of Heart, Lung and Blood Vessel Diseases, Beijing, China

**Keywords:** hybrid, stented elephant trunk, thoracic endovascular aortic repair, extra-anatomic bypass, distal arch

## Abstract

**Objective:** This study aims to compare the short- and mid-term outcomes of the stented elephant trunk (SET) procedure combined with supra-arch branch reconstruction and one-stage hybrid arch repair combined thoracic endovascular aortic repair (TEVAR) with extra-anatomic bypass in the management of distal arch disease.

**Methods:** From January 2009 to January 2019, 97 patients underwent one-stage hybrid arch repair combined with TEVAR with extra-anatomic bypass (HAR group), and 206 patients underwent the SET procedure with supra-arch branch reconstruction (SET group). We used inverse-probability-of treatment weighting (IPTW) to adjust baseline differences.

**Results:** Before IPTW adjustment, there was no significant difference in operative mortality between the two groups (5.2 vs. 1.0%, *P* = 0.064). The incidences of stroke, spinal cord injury (SCI), acute kidney injury (AKI), and endoleak also showed no significant differences (4.1 vs. 0.5%, *P* = 0.066; 2.1 vs. 1.5%, *P* = 1.000; 0 vs. 1.0%, *P* = 0.831; 6.2 vs. 1.9%, *P* = 0.113, respectively). After IPTW adjustment, the incidences of stroke, SCI, and AKI showed no significant differences between the two groups (1.8 vs. 1.1%, *P* = 0.138; 0.8 vs. 1.6%, *P* = 0.448; and 0 vs. 0.7%, *P* = 0.148, respectively). However, the HAR group tended to have higher operative mortality and incidence of endoleak than the SET group (12.4 vs. 1.3%, *P* = 0.01; 9.9 vs. 1.8%, *P* = 0.031, respectively). In the multivariate analysis, open repair decreased the risks of endoleak (odds ratio [OR], 0.171, 95% CI, 0.060–0.401; *P* < 0.001) and operative mortality (OR, 0.093, 95% CI, 0.027–0.238; *P* < 0.001). The overall survival and event-free survival of the HAR group were significantly lower than those of the SET group (*P* < 0.001).

**Conclusion:** One-stage hybrid arch repair combined TEVAR with extra-anatomic bypass and the SET procedure with supra-arch branch reconstruction both provided good postoperative treatment outcomes for distal arch disease. However, hybrid arch repair increased the risks of endoleak and operative mortality. The SET procedure provided better mid-term survival than hybrid arch repair without increasing operative mortality. Carefully selecting the indications for the procedure, while receiving close long-term follow-up, may improve the survival rate of patients undergoing hybrid arch repair.

## Introduction

Thoracic endovascular aortic repair (TEVAR) combined with extra-anatomic bypass is a minimally invasive technique enabling to overcome the coverage of supra-arch branch (1). This hybrid procedure extends the indications for endovascular treatment, and is gradually being used to treat distal aortic arch disease (2). Although open arch repair correlated with higher perioperative mortality and complications (3), our previous studies demonstrated that open arch repair combined with supra-arch branch reconstruction also achieved excellent results to manage distal arch disease (4, 5). Currently, the hybrid procedure serves as an alternative management option for individuals who are unsuitable for open repair (6). Whether the hybrid procedure can replace conventional open repair also remains controversial (6, 7). Moreover, no study objectively compared the treatment outcomes of the aforementioned approaches. We attempt to make a comparison between one-stage hybrid arch repair combined TEVAR with extra-anatomic bypass and the stented elephant trunk (SET) procedure combined with supra-arch branches reconstruction for the short- and mid-term effectiveness in treating distal aortic arch disease at our institution using inverse probability treatment weighting (IPTW).

## Methods

### Patients

**Figure 1:**
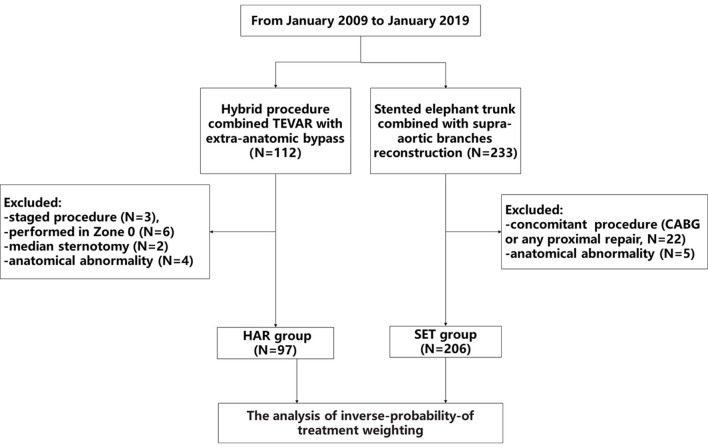
The detailed flow chart of inclusion.

Between January 2009 and January 2019, one-stage hybrid arch repair combined TEVAR with extra-anatomic bypass (HAR group) were performed in 112 patients, and the SET procedure combined with supra-arch branch reconstruction (SET group) were performed in 233 patients at Beijing Anzhen Hospital. Patients with anatomical abnormalities, as well as those who underwent a staged procedure, the Zone 0 hybrid procedure, or concomitant proximal repair were excluded. As a result, 97 and 206 individuals were included in the HAR and SET groups, respectively. A detailed flow chart showing study inclusion is shown in Figure 1. A multidisciplinary team decided whether to perform the hybrid or open procedure, and the specific evaluation factors have been described in detail in our previously study (8). Since the hybrid extra-anatomic technique is used to treat high-risk patients, the patient backgrounds may be different between the HAR and SET groups. We used IPTW to adjust the baseline differences between the two groups. The Ethics Committees of Beijing Anzhen Hospital of Capital Medical University approved this retrospective study (No. 2019059X), which waived the requirement for informed consent because of the retrospective nature of the analysis.

### Hybrid Procedure

**Figure 2:**
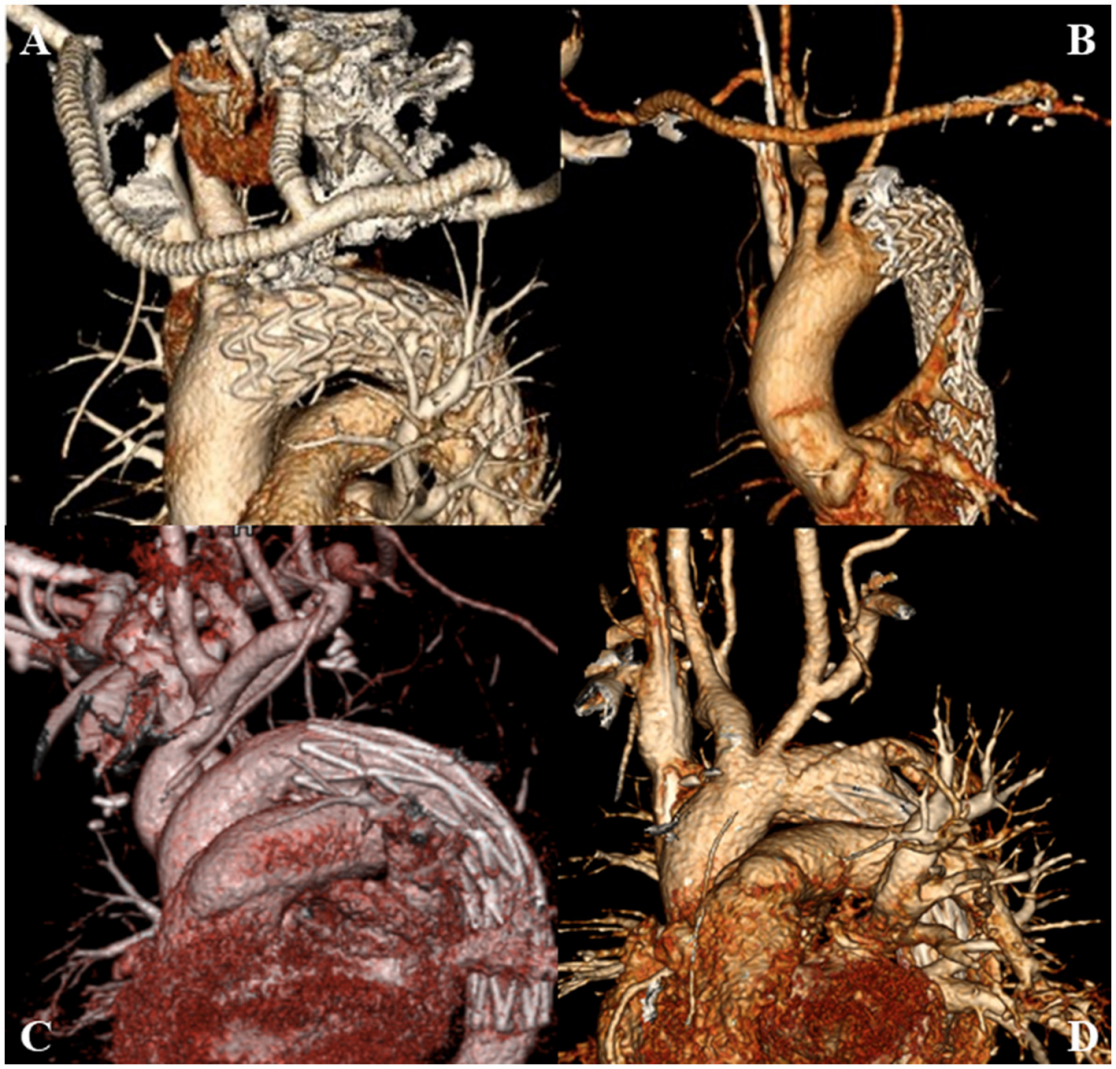
**(A)** HAR performed in Zone 1; **(B)** HAR performed in Zone 2; **(C)** SET performed in Zone 1; **(D)** SET performed in Zone 2. SET, stent elephant trunk procedure combined with supra-aortic branch reconstruction; HAR, hybrid procedure combined TEVAR with extra-anatomic bypass.

In Zone 1, the bilateral axillary arteries were dissociated through subclavian incisions, and the left common carotid artery (LCCA) was dissociated through the anterior edge of the sternocleidomastoid muscle. The bypass from the right axillary artery (RAA) to the LCCA and left axillary artery (LAA) was established with a bifurcate “T” GORE-TEX^®^ (W.L. Gore and Ass., Flagstaff, AZ, USA) prosthetic graft. The proximal end of the LCCA was ligated. In Zone 2, only the extra-anatomic bypass from the RAA to the LAA was established with a straight GORE-TEX^®^ prosthetic graft. A transfemoral TEVAR procedure was initiated after the establishment of the extra-anatomic bypass (8, 9). The diameter of the aortic arch was measured again by aortic angiography. The final diameter of the arch was determined by comparing the two measurements. The oversizing of the stent-grafts was not more than 10–15%, and the proximal landing zone needed to be more than 20 mm. Finally, the proximal end of the LSCA was embolized via the LAA (Figures 2A,B).

### Open Repair

The surgical technique was previously described (4). Briefly, all patients underwent a median sternotomy under cardiopulmonary bypass (CPB) with selective antegrade cerebral perfusion (SACP) under general anesthesia. The supra-arch branches were exposed. After heparinization (3 mg/kg), CPB was established via the RAA and the right atrium or the superior and inferior vena cava. Perfusion via cold blood cardioplegia arrested the heart. When the nasopharyngeal temperature reached 25°C, SACP was initiated (5–10 mL/kg/min). The distal aortic arch was longitudinally incised between the origin of the LCCA and the origin of the LSCA. A self-expandable stented graft (MicroPort, Shanghai, China) was inserted into the true lumen and anastomosed on the normal aortic wall in a continuous circumferential full-thickness manner. Then, CPB gradually resumed to normal flow, and the rewarming process began. The LSCA was transected circumferentially at 5–10 mm distal to its origin, and the proximal end was ligated. The distal end was anastomosed end-to-side to the LCCA in a continuous manner. If the LSCA and LCCA needed to be reconstructed, they were anastomosed with prosthetic grafts to the normal aortic wall. When the nasopharyngeal temperature reached 36.5°C, the patient was weaned from CPB (Figures 2C,D).

### Definitions and Follow-Up

Operative mortality was defined as death within 30 days after surgery or during the same hospitalization following surgical treatment, even if it occurred after 30 days. Previous cardiac/aortic procedures were defined as a history of open or interventional therapy of the heart or aorta. Stroke was defined as any new global or focal neurological deficit lasting ≥24 h with an acute lesion on brain imaging. Spinal cord injury (SCI) was defined as any new lower extremity deficit that was unrelated to an intracerebral event. If patients developed SCI, cerebrospinal fluid drainage was performed. Acute kidney injury (AKI) was defined as a rise in serum creatinine of 1 mg/dL above baseline or a new requirement for renal dialysis (8, 10). The zones of the arch were defined following the Ishimaru classification (11). The follow-up was accomplished by telephone and hospital chat. The recommendation for all patients after discharge was to perform CTA at month 6, month 12, and annually thereafter.

### Statistics

Continuous variables with normal distribution were expressed by mean ± standard deviation, and *t*-tests were used for comparison of the variables. Otherwise, the median (interquartile range, IQR) was reported and the Mann-Whitney *U* test was used for comparison of the variables. Categorical data are presented as numbers (percentages) and were compared using Chi-square or Fisher's exact tests, where appropriate. The Kaplan-Meier model was used to analyze overall survival and event-free survival, and the differences were compared by a log-rank test. Multivariate stepwise regression analysis based on the Akaike information criterion was used to determine the risk factors. We used IPTW to adjust baseline differences between the two groups. The covariates included age, sex, hypertension, chronic obstructive pulmonary disease, coronary artery disease, diabetes, previous cerebrovascular disease, previous cardiac/aortic procedures, renal insufficiency, aortic dissection, and aneurysm. Statistical significance was considered as *P* < 0.05 with two-sided hypothesis testing. All statistics were analyzed using R for Windows version 3.3.3 (The R Foundation for Statistical Computing, Vienna, Austria).

## Results

### Patient Characteristics

**Figure 3:**
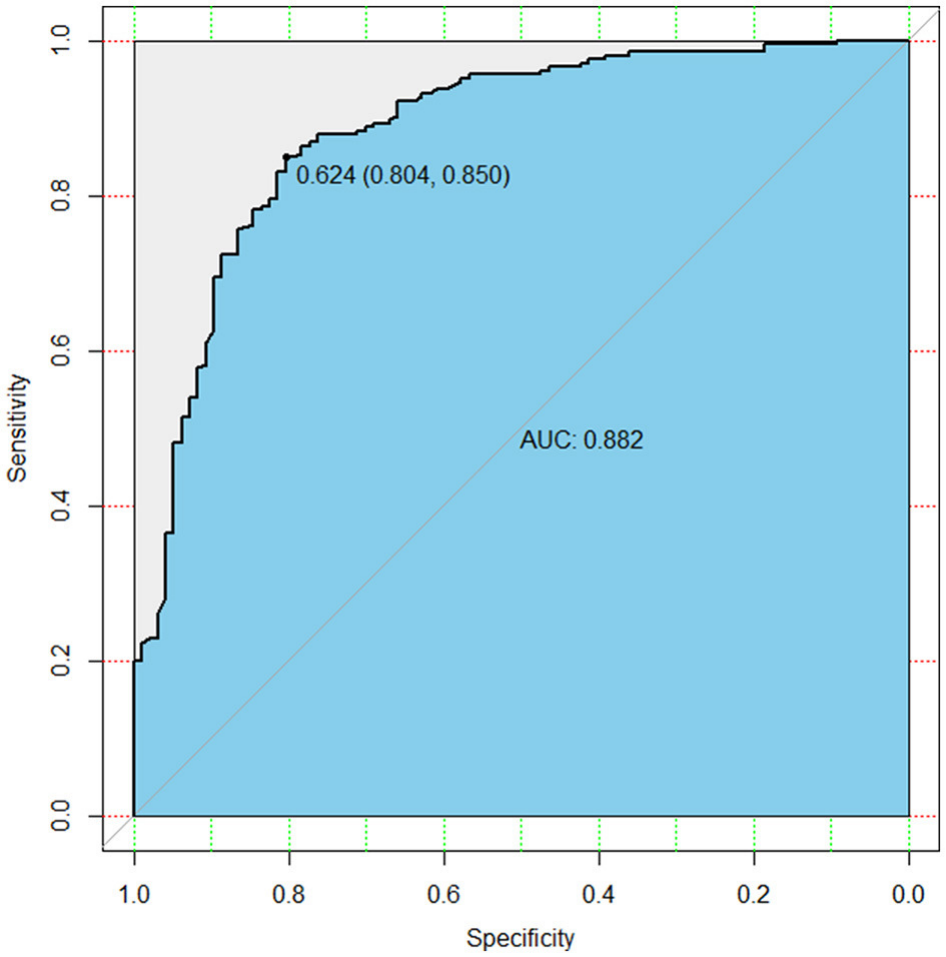
The risk-adjusted model showed good discriminatory power (C-statistic = 0.882).

Mean age differed significantly between the two groups (62.5 ± 9.0 vs. 47.5 ± 10.3, *P* < 0.001). Coronary artery disease, diabetes and previous heart/aortic surgery were more prevalent in the HAR group in comparison to the SET group (22.7% [22/97] vs. 4.4% [9/206], *P* < 0.001;15.5% [15/97] vs. 5.3% [11/206], *P* = 0.007; 21.6% [21/97] vs. 8.7% [18/206], *P* = 0.003, respectively). The prevalence of previous cerebrovascular disease was also higher in the HAR group (12.4% [12/97] vs. 4.4% [9/206], *P* = 0.021) (Table 1). The baseline differences between the two groups were adjusted using IPTW. The baseline comparisons did not differ after adjustment (Table 1). The discriminatory power of the risk-adjusted model was presented in Figure 3 (C-statistic = 0.882).

**Table 1:** Baseline before and after IPTW.

**Variables**	**Unadjusted data**			**Data adjusted by IPTW**		
	**Hybrid (*n* = 97)**	**Open (*n* = 206)**	***P*-value**	**Hybrid (*n* = 97)**	**Open (*n* = 206)**	***P*-value**
Sex (male)	84 (86.6%)	181 (87.9%)	0.901	83.9%	86.1%	0.779
Age (years)	62.5 ± 9.0	47.5 ± 10.3	<0.001	51.7 ± 11.7	51.8 ± 11.5	0.998
Hypertension	77 (79.4%)	150 (72.8%)	0.277	75.0%	75.1%	0.996
CAD	22 (22.7%)	9 (4.4%)	<0.001	8.5%	9.5%	0.834
Diabetes	15 (15.5%)	11 (5.3%)	0.007	6.1%	8.0%	0.588
COPD	2 (2.1%)	2 (1.0%)	0.813	0.7%	0.8%	0.939
Renal insufficiency	3 (3.1%)	6 (2.9%)	1.000	1.8%	2.5%	0.661
Previous cerebrovascular disease	12 (12.4%)	9 (4.4%)	0.021	7.8%	6.5%	0.793
Previous cardiac/aortic procedures	21 (21.6%)	18 (8.7%)	0.003	17.5%	13.5%	0.571
Dissection	31 (32.0%)	155 (75.2%)	<0.001	61.9%	62.0%	0.987
Aneurysm	31 (32.0%)	17 (8.3%)	<0.001	20.1%	17.2%	0.714

*CAD, cardiac artery disease; COPD, chronic obstructive pulmonary disease; IPTW, inverse probability of treatment weighting*.

### Short-Term Outcomes

Before IPTW, the operative time was markedly shorter in the HAR group (316.3 ± 80.8 vs. 340.3 ± 55.5, *P* = 0.003). Although the HAR group had shorter times of ICU stay and mechanical ventilation than the SET group, the comparisons were not significant (29.7 ± 63.2 vs. 32.9 ± 48.5, *P* = 0.634; 19.1 ± 67.3 vs. 20.5 ± 12.1, *P* = 0.774, respectively). The median hospitalization time and operative mortality did not differ significantly (17.0 ± 9.8 vs. 15.9 ± 7.0, *P* = 0.252; 5.2% [5/97] vs. 1.0% [2/206], *P* = 0.064). In addition, complications including stroke, spinal cord injury, AKI, and endoleaks failed to differ between the two groups (4.1% [4/97] vs. 0.5% [1/206], *P* = 0.066; 2.1% [2/97] vs. 1.5% [3/206], *P* = 1.000; 0% [0/97] vs. 1.0% [2/206], *P* = 0.831; 6.2% [6/97] vs. 1.9% [2/206], *P* = 0.113, respectively) (Table 2).

**Table 2:** Postoperative outcomes comparisons before and after IPTW.

**Variables**	**Unadjusted data**			**Data adjusted by IPTW**		
	**Hybrid (*n* = 97)**	**Open (*n* = 206)**	***P*-value**	**Hybrid (*n* = 97)**	**Open (*n* = 206)**	***P*-value**
Hospitalization (d)	17.0 ± 9.8	15.9 ± 7.0	0.252	15.5 ± 7.4	17.0 ± 7.7	0.217
Operation time (min)	316.3 ± 80.8	340.3 ± 55.5	0.003	331.0 ± 80.2	343.7 ± 54.5	0.455
ICU stay (h)	29.7 ± 63.2	32.9 ± 48.5	0.634	62.4 ± 113.9	33.85 ± 46.4	0.289
MV (h)	19.1 ± 67.3	20.5 ± 12.1	0.774	21.4 ± 49.7	20.7 ± 11.9	0.947
Stroke	4 (4.1%)	1 (0.5%)	0.066	1.8%	1.1%	0.138
Spinal cord injury	2 (2.1%)	3 (1.5%)	1.000	0.8%	1.6%	0.448
AKI	0 (0.0%)	2 (1.0%)	0.831	0.0%	0.7%	0.148
Endoleak	6 (6.2%)	4 (1.9%)	0.113	9.9%	1.8%	0.031
Operative mortality	5 (5.2%)	2 (1.0%)	0.064	12.4%	1.3%	0.010

*MV, mechanical ventilation; AKI, acute kidney injury; IPTW, inverse probability of treatment weighting*.

After IPTW risk-adjusted analysis, hospitalization, ICU stay, or mechanical ventilation time did not differ between the two groups. Complication rates such as stroke, SCI, and AKI were similarly not different between the two groups (1.8 vs. 1.1%, *P* = 0.138; 0.8 vs. 1.6%, *P* = 0.448; and 0 vs. 0.7%, *P* = 0.148, respectively). However, operative mortality and the incidence of endoleak were statistically higher in the HAR group than in the SET group (12.4 vs. 1.3%, *P* = 0.01; 9.9 vs. 1.8%, *P* = 0.031, respectively) (Table 2).

### Mid-term Outcomes

Average follow-up was 38.8 ± 26.6 months (range, 1.5–130.8 months; median 39.7 months). During the follow-up period, there was one death from stroke, one death from severe pneumonia, three deaths from aortic dissection, and nine sudden deaths in the HAR group. Late endoleaks occurred in six individuals (type I = 5, type II = 1). Three other patients developed late complications. One patient had a new entry due to a distal stent graft. Another patient developed a slight stenosis in the extra-anatomic bypass with no indication for re-intervention. A further patient presented with arm numbness without evidence of any neurological injury or ischemia.

Six deaths occurred in the SET group during the follow-up; these were caused by a severe pulmonary infection (*n* = 1), respiratory failure after an additional ascending aorta and total aortic arch replacement concomitant with the Wheat procedure (*n* = 1), circulatory failure after an additional total aortic arch replacement (*n* = 1), and sudden death (*n* = 3). Four patients underwent additional endovascular repair. The causes included one new abdominal aortic lesion, one new penetrating ulcer at the distal end of the SET, two anatomic leakages. An additional three patients survived after undergoing a secondary open repair. One patient underwent ascending aorta replacement because of a new ascending aortic dissection. Two patients underwent planned second-stage thoracoabdominal aortic replacement.

**Figure 4:**
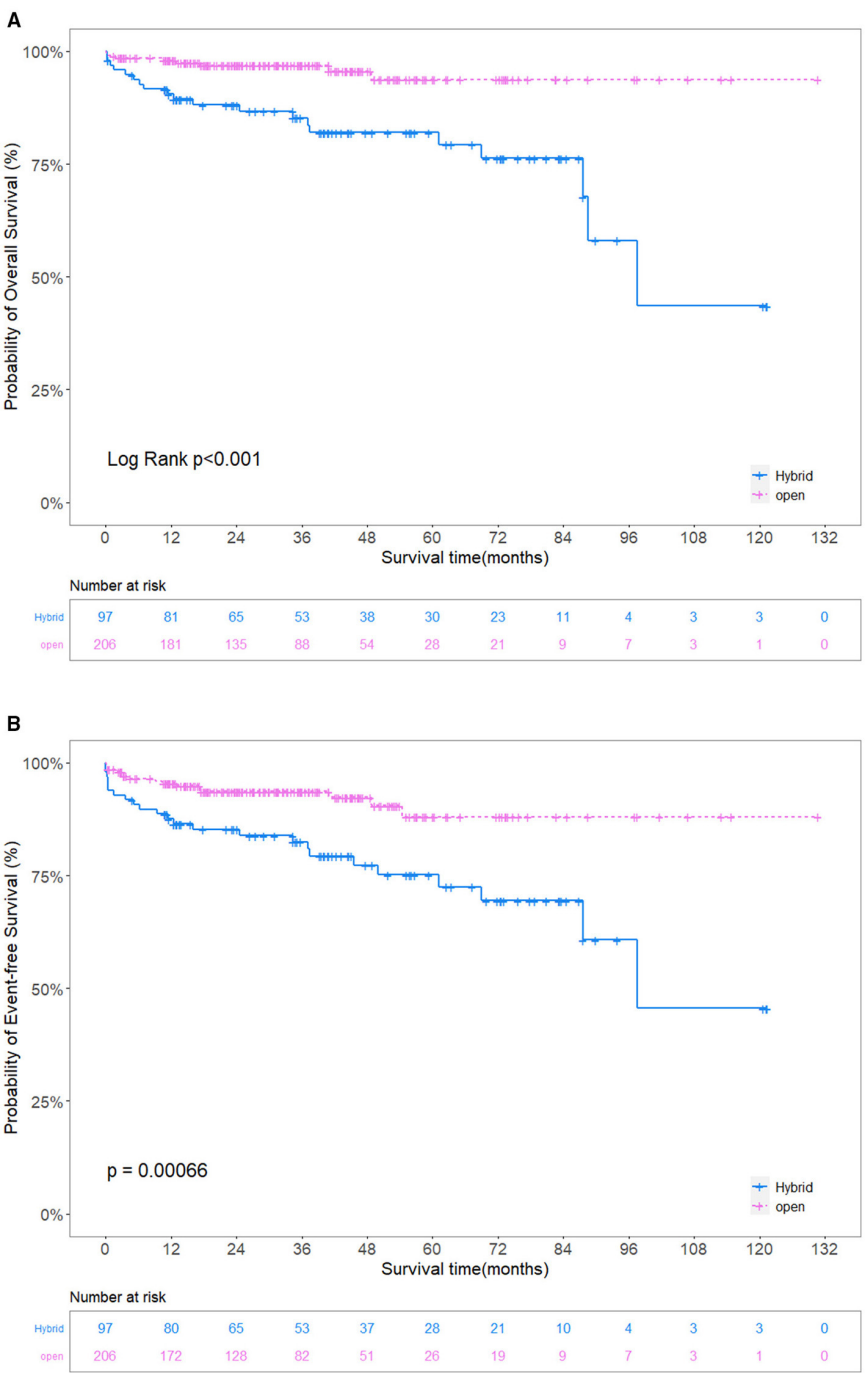
**(A)** Unadjusted comparison of the overall survival. **(B)** Unadjusted comparison of the event-free survival. The overall survival and event-free survival in the SET group were significantly better than those in the HAR group. SET, stent elephant trunk procedure combined with supra-aortic branch reconstruction; HAR, hybrid procedure combined TEVAR with extra-anatomic bypass.

**Figure 5:**
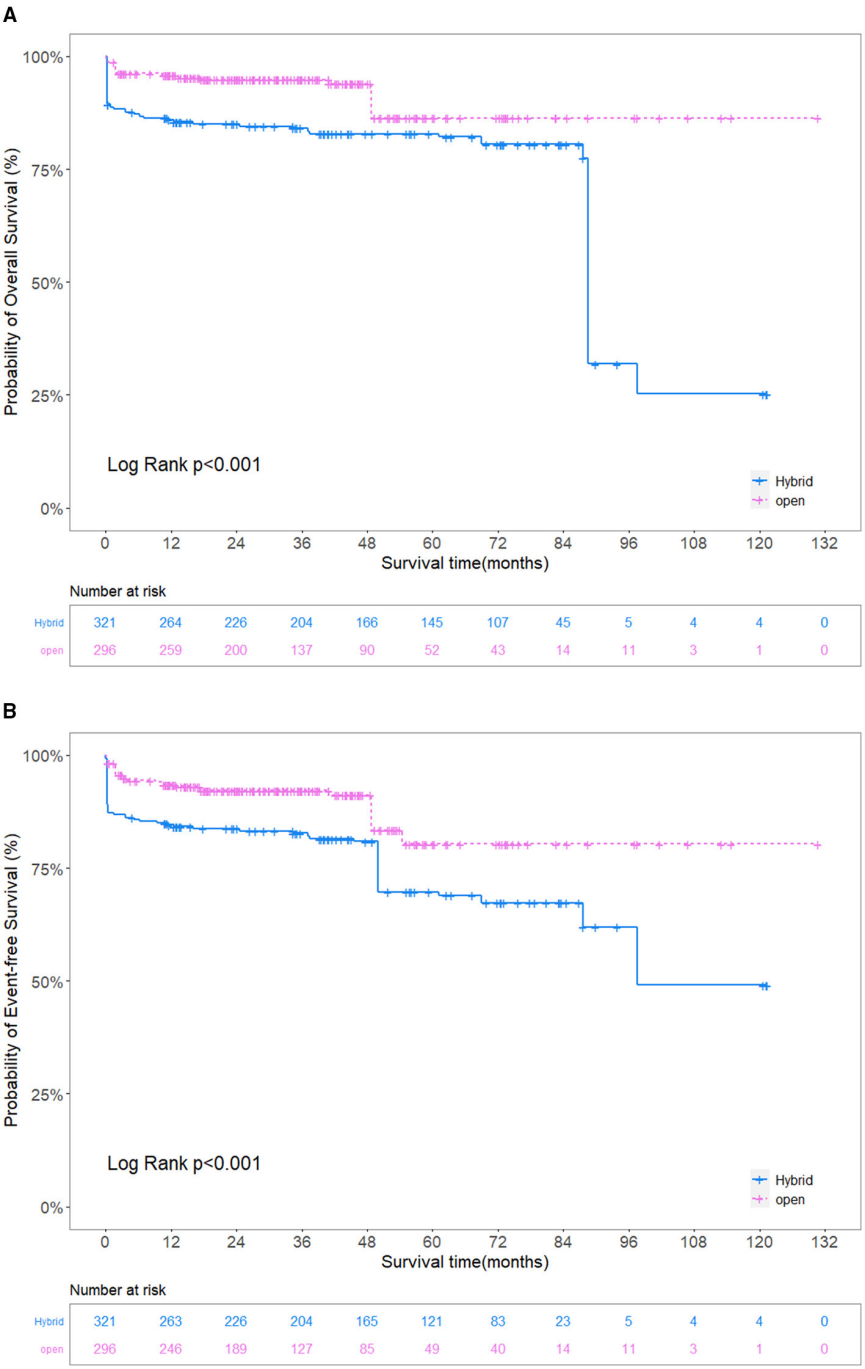
**(A)** Adjusted comparison of the overall survival. **(B)** Adjusted comparison of the event-free survival. The overall survival and event-free survival in the SET group were significantly better than those in the HAR group. SET, stent elephant trunk procedure combined with supra-aortic branch reconstruction; HAR, hybrid procedure combined TEVAR with extra-anatomic bypass.

The overall survival in the HAR group was 90.5% (95% confidence interval [CI] 84.8–96.6%), 82.0% (95% CI 74.0–90.9%) and 43.6% (21.7–87.4%) at 1, 5, and 9 years, respectively. The overall survival in the SET group was 98.0% at 1 year (95% CI 96.1–100%) and 93.8% (95% CI 88.9–98.8%) at 5–9 years. Overall survival and event-free survival differed significantly (*P* < 0.001) (Figure 4). IPTW adjustment also yielded the same results (*P* < 0.001) (Figure 5). Patients in the SET group attained significantly lower risks of endoleak (odds ratio [OR], 0.171, 95% CI, 0.060–0.401; *P* < 0.001) and operative death (OR, 0.093, 95% CI, 0.027–0.238; *P* < 0.001) than those in the HAR group during the multivariate analysis (Table 3).

**Table 3:** AIC stepwise regression analysis of postoperative complications of SET group vs. HAR group (adjusted by IPTW).

	**Estimate**	**OR**	**95%CI**		***P*-value**
			**Lower**	**Upper**	
**Variables of multivariate analysis**					
Endoleak	−1.768	0.171	0.060	0.401	<0.001
Operative death	−2.376	0.093	0.027	0.238	<0.001
**Variables of univariate analysis**					
Stroke	−1.563	0.210	0.014	1.171	0.128
Spinal cord injury	0.730	2.075	0.471	11.740	0.350
Endoleak	−1.763	0.172	0.061	0.399	<0.001
Operative death	−2.373	0.093	0.027	0.238	<0.001

*IPTW, inverse probability of treatment weighting*.

## Comment

The hybrid procedure is a less invasive approach combining open and endovascular repair, and is still considered an alternative management option to open repair due to controversial treatment outcomes (12). Our results revealed that hybrid arch repair did not significantly reduce hospitalization, ICU stay, or mechanical ventilation time. Compared with the SET group, although hybrid arch repair did not increase the incidences of stroke, SCI, and AKI, it increased operative mortality and the incidence of endoleak. Meanwhile, based on the multivariate analysis, the HAR group tended to have significantly higher risks of endoleak and operative mortality than the SET group. The SET group achieved significant superiority over the HAR group in terms of overall survival and event-free survival. This study suggested that the SET procedure combined with supra-arch branch reconstruction could provide safer and more reliable treatment outcomes.

Hybrid arch repair led to high mortality and the incidence of neurological complications (13). Joo et al. (14) argued that hybrid arch repair only reduced the risk of lung complications and did not improve the mid-term survival of patients. Another risk-adjusted study demonstrated that hybrid arch repair was not superior to open arch repair. Their results revealed that the hybrid procedure only shortened ICU stay and hospitalization (15). Our study yielded even more pessimistic results. Hybrid arch repair did not show a significant advantage over open repair including hospitalization, ICU stay, and mechanical ventilation time. Moreover, the SET group also showed better overall survival and event-free survival than the HAR group. According to our analysis, a proportion of deaths in the HAR group were not only caused by aortic events but also by other procedure-related complications. In addition, the two groups present some differences in the causes of aortic events. Aortic events occurred in the SET group mainly due to new aortic lesions rather than the initial procedure, while aortic events in the HAR group were mostly related to the initial hybrid procedure.

The American College of Surgeons suggested that the risk of stroke was twice as high with extra-anatomic bypass than with transposition (16). Stroke was considered a serious postoperative complication, the incidence of which was reported to range from 0 to 28.6% in the literature (13, 17). Although our results revealed a low incidence of stroke, in total, four patients died of this complication. Currently, there was a strong relationship between hemodynamic hypoperfusion and stroke (18). Additionally, TEVAR-related operations such as wires and delivery system placement were also related to the occurrence of stroke (19–21). Conversely, SACP is considered to reduce the chance of post-operative stroke (22), it maintained stable intraoperative blood pressure and cerebral perfusion (23). Moreover, anterograde stented graft implantation under direct vision made it easier to confirm stent release in the true lumen, which also avoided the possibility of stroke. Although our results failed to show that hybrid arch repair increased the incidence of stroke, some surgeons avoided the neurological complications by establishing cerebral perfusion during hybrid arch repair. Their short-term follow-up results also confirmed the superiority of continuous and stable brain perfusion in reducing neurological complications (24, 25). Therefore, a shorter hypothermic circulatory arrest time combined with stable cerebral perfusion could contribute to the reduction in the occurrence of stroke.

Yasuhisa and colleagues (26) argued that aortic lesions coexisting with inadequate landing zones, severe aortic atherosclerosis, or large arterial diameters should not be treated with TEVAR. We suggest that an adequate proximal and distal landing zone is essential to avoid endoleak, since aortic lesions will be completely covered. In addition, the strategy of stenting should be based on the pathologies and prognoses of individuals. In our center, for dissection involving the distal arch, we performed two-stent implantation with tapered stents to avoid endoleak (27). Notably, late reintervention may be unavoidable for degenerative aortic lesions. Therefore, such patients should receive long-term follow-up.

Canaud et al. (28) treated 11 patients using sequential transposition of supra-arch branches. No permanent cerebral or SCI was observed during the follow-up. Our study provided treatment outcomes for a larger cohort and demonstrated the effectiveness of open supra-arch branch reconstruction. SET implantation not only provided a reliable proximal landing zone for reintervention or planned second-stage procedures, but also avoided covering more normal aortic segments. Meanwhile, the continuous full-thickness suturing method also reduced the incidence of anastomotic leakage (4, 29). In the SET group, two patients underwent planned second-stage thoracic and abdominal aortic replacement. They recovered well after the operation and were discharged without any adverse events. Although the other two patients died from secondary extended repair of the proximal aortic arch, this result might pertain to the patient's unfavorable preoperative status and did not indicate that the open repair increased the risk of death from reintervention.

### Study Limitations

The current study has several limitations. Although we applied IPTW to adjusted for the impact of selection bias, the results of the entire cohort may still be influenced by single-center retrospective nature and different pathologies of aortic arch lesions. Furthermore, this study compared only the results of one-stage hybrid arch repair combined TEVAR with extra-anatomic bypass and the SET procedure combined with supra-arch reconstruction; thus, this result could not extrapolate to prove that SET combined with supra-arch reconstruction is superior to other hybrid or endoluminal repair techniques.

## Conclusion

These risk-adjusted results revealed that one-stage hybrid arch repair combined TEVAR with extra-anatomic bypass and the SET procedure with supra-arch branch reconstruction both provided good postoperative treatment outcomes for distal arch disease. However, hybrid arch repair increased the risks of endoleak and operative mortality. The SET procedure provided better mid-term survival than hybrid arch repair without increasing operative mortality. Carefully selecting the indications for the procedure, while receiving close long-term follow-up, may improve the survival rate of patients undergoing hybrid arch repair.

## Data Availability Statement

The original contributions generated for this study are included in the article/supplementary material, further inquiries can be directed to the corresponding author/s.

## Ethics Statement

The studies involving human participants were reviewed and approved by The Ethics Committees of Beijing Anzhen Hospital of Capital Medical University. Written informed consent for participation was not required for this study in accordance with the national legislation and the institutional requirements.

## Author Contributions

ZQ, SC, JZ, and LS designed the study and wrote the initial manuscript. ZQ, SC, YG, and CL conducted data collection and searched the literature. ZQ, SC, RG, and YZ collated the data and performed statistical analysis. JZ, YL, and LS revised and reviewed the manuscript. All authors contributed to the submitted version and agreed to be responsible for the content of the work.

## Conflict of Interest

The authors declare that the research was conducted in the absence of any commercial or financial relationships that could be construed as a potential conflict of interest.

## Publisher's Note

All claims expressed in this article are solely those of the authors and do not necessarily represent those of their affiliated organizations, or those of the publisher, the editors and the reviewers. Any product that may be evaluated in this article, or claim that may be made by its manufacturer, is not guaranteed or endorsed by the publisher.

## References

[1] Lee WA Matsumura JS Mitchell RS Farber MA Greenberg RK Azizzadeh A. Endovascular repair of traumatic thoracic aortic injury: clinical practice guidelines of the Society for Vascular Surgery. J Vasc Surg. (2011) 53:187–92. 10.1016/j.jvs.2010.08.027 20974523

[2] Ishibashi H Ishiguchi T Ohta T Sugimoto I Iwata H Yamada T. Partial debranching hybrid stent graft for distal aortic arch aneurysms. Surg Today. (2012) 42:765–9. 10.1007/s00595-012-0139-3 22318637

[3] Tsai TT Isselbacher EM Trimarchi S Bossone E Pape L Januzzi JL. Acute type B aortic dissection: does aortic arch involvement affect management and outcomes? Insights from the International Registry of Acute Aortic Dissection (IRAD). Circulation. (2007) 116:I150–6. 10.1161/CIRCULATIONAHA.106.681510 17846296

[4] Zhu J-M Qi R-D Chen L Liu W Li C-N Fan Z-M. Stented elephant trunk procedure with left subclavian artery transposition for acute type B dissection with distal arch involvement. J Thorac Cardiovasc Surg. (2015) 150:1160–5. 10.1016/j.jtcvs.2015.07.089 26344688

[5] Qi RD Zhu JM Liu YM Chen L Li CN Xing XY. Distal arch aneurysm repair using left subclavian artery transposition with stented elephant trunk in the hybrid repair era. Heart Lung Circ. (2019) 28:814–9. 10.1016/j.hlc.2018.03.014 29685718

[6] Tokuda Y Oshima H Narita Y Abe T Araki Y Mutsuga M. Hybrid versus open repair of aortic arch aneurysms: comparison of postoperative and mid-term outcomes with a propensity score-matching analysis. Eur J Cardiothorac Surg. (2016) 49:149–56. 10.1093/ejcts/ezv063 25732968

[7] Furutachi A Takamatsu M Nogami E Hamada K Yunoki J Itoh M. Early and mid-term outcomes of total arch replacement with the frozen elephant trunk technique for type A acute aortic dissection. Interact Cardiovasc Thorac Surg. (2019) 29:753–60. 10.1093/icvts/ivz154 31230069

[8] Chen SW Zhong YL Qiao ZY Li CN Ge YP Qi RD. One-stage hybrid procedure for distal aortic arch disease: mid-term experience at a single center. J Thorac Dis. (2020) 12:7117–26. 10.21037/jtd-20-2338 33447400 PMC7797852

[9] Xu SD Huang FJ Yang JF Li ZZ Yang S Du JH. Early and midterm results of thoracic endovascular aortic repair of chronic type B aortic dissection. J Thorac Cardiovasc Surg. (2010) 139:1548–53. 10.1016/j.jtcvs.2009.08.051 19910003

[10] Ma WG Zhu JM Zhang W Sun K Ziganshin BA Wang L-F. Frozen elephant trunk with total arch replacement for type A aortic dissections: does acuity affect operative mortality? J Thorac Cardiovasc Surg. (2014) 148:963–70. 10.1016/j.jtcvs.2014.06.005 25043867

[11] Fillinger MF Greenberg RK McKinsey JF Chaikof EL Society for Vascular Surgery Ad Hoc Committee on TRS. Reporting standards for thoracic endovascular aortic repair (TEVAR). J Vasc Surg. (2010) 52:1022–33. 10.1016/j.jvs.2010.07.008 20888533

[12] Faure EM Canaud L Marty-Ane C Alric P. Hybrid aortic arch repair for dissecting aneurysm. J Thorac Cardiovasc Surg. (2016) 152:162–8. 10.1016/j.jtcvs.2016.03.020 27068438

[13] Cao P De Rango P Czerny M Evangelista A Fattori R Nienaber C. Systematic review of clinical outcomes in hybrid procedures for aortic arch dissections and other arch diseases. J Thorac Cardiovasc Surg. (2012) 144:1286–300. 10.1016/j.jtcvs.2012.06.013 22789301

[14] Joo HC Youn YN Ko YG Choi D Won JY Lee DY. Comparison of open surgical versus hybrid endovascular repair for descending thoracic aortic aneurysms with distal arch involvement. J Thorac Dis. (2018) 10:3548–57. 10.21037/jtd.2018.05.127 30069352 PMC6051785

[15] Iba Y Minatoya K Matsuda H Sasaki H Tanaka H Oda T. How should aortic arch aneurysms be treated in the endovascular aortic repair era? A risk-adjusted comparison between open and hybrid arch repair using propensity score-matching analysis. Eur J Cardiothorac Surg. (2014) 46:32–9. 10.1093/ejcts/ezt615 24431168

[16] Madenci AL Ozaki CK Belkin M McPhee JT. Carotid-subclavian bypass and subclavian-carotid transposition in the thoracic endovascular aortic repair era. J Vasc Surg. (2013) 57:1275–82.e2. 10.1016/j.jvs.2012.11.044 23384492

[17] Hiraoka A Chikazawa G Tamura K Totsugawa T Sakaguchi T Yoshitaka H. Clinical outcomes of different approaches to aortic arch disease. J Vasc Surg. (2015) 61:88–95. 10.1016/j.jvs.2014.06.121 25095745

[18] Bibiloni Lage I Calsina Juscafresa L Delgado Dominguez C Bilbao Jaureguizar JI Bastarrika G Rabago Juan-Aracil G. Hybrid repair of aortic arch aneurysms with endografting of the ascending aorta. J Card Surg. (2016) 31:341–7. 10.1111/jocs.12735 27005830

[19] Hiraoka A Chikazawa G Totsugawa T Tamura K Ishida A Sakaguchi T. Objective analysis of midterm outcomes of conventional and hybrid aortic arch repair by propensity-score matching. J Thorac Cardiovasc Surg. (2017) 154:100–106.e1. 10.1016/j.jtcvs.2016.12.060 28314530

[20] Bunger CM Kische S Liebold A Leissner M Glass A Schareck W. Hybrid aortic arch repair for complicated type B aortic dissection. J Vasc Surg. (2013) 58:1490–6. 10.1016/j.jvs.2013.05.091 23880549

[21] Lotfi S Clough RE Ali T Salter R Young CP Bell R. Hybrid repair of complex thoracic aortic arch pathology: long-term outcomes of extra-anatomic bypass grafting of the supra-aortic trunk. Cardiovasc Intervent Radiol. (2013) 36:46–55. 10.1007/s00270-012-0383-3 22526104

[22] Misfeld M Leontyev S Borger MA Gindensperger O Lehmann S Legare JF. What is the best strategy for brain protection in patients undergoing aortic arch surgery? A single center experience of 636 patients. Ann Thorac Surg. (2012) 93:1502–8. 10.1016/j.athoracsur.2012.01.106 22480393

[23] Englum BR Andersen ND Husain AM Mathew JP Hughes GC. Degree of hypothermia in aortic arch surgery - optimal temperature for cerebral and spinal protection: deep hypothermia remains the gold standard in the absence of randomized data. Ann Cardiothorac Surg. (2013) 2:184–93. 10.3978/j.issn.2225-319X.2013.03.01 23977581 PMC3741845

[24] Oztas DM Ugurlucan M Beyaz MO Ulukan MO Unal O Onal Y. Follow-up results of aortic arch cervical debranching performed with the help of a temporary crossover external carotid artery bypass for cerebral protection followed by endovascular thoracic aortic aneurysm repair. Interact Cardiovasc Thorac Surg. (2020) 30:724–31. 10.1093/icvts/ivaa004 32073125

[25] Ugurlucan M Sayin OA Onalan MA Alishev N Basaran M Alpagut U. Cerebral protection with a crossover external carotid artery bypass during arch debranching. Ann Thorac Surg. (2015) 99:725–7. 10.1016/j.athoracsur.2014.07.089 25639427

[26] Oishi Y Sonoda H Ushijima T Kimura S Tatewaki H Tanoue Y. Single-stage hybrid total arch replacement for extended arch aneurysms. J Vasc Surg. (2019) 69:1719–25. 10.1016/j.jvs.2018.08.184 31159980

[27] Gao HQ Xu SD Ren CW Yang S Liu CL Zhen J. Analysis of perioperative outcome and long-term survival rate of thoracic endovascular aortic repair in uncomplicated type B dissection: single-centre experience with 751 patients. Eur J Cardiothorac Surg. (2019) 56:1090–6. 10.1093/ejcts/ezz131 31329842

[28] Canaud L Joyeux F Ziza V Branchereau P Marty-Ane C Alric P. Hemi-aortic arch debranching for hybrid aortic arch repair by sequential transposition of the left common carotid and subclavian arteries. J Thorac Cardiovasc Surg. (2013) 145:764–7. 10.1016/j.jtcvs.2012.03.012 22480885

[29] Zhu J Cheng L Liu Y Zheng J Qiao Z Li C. One-stage repair for stanford type B aortic dissection concomitant with cardiac diseases: open stented elephant trunk technique combined with cardiac operation. Thorac Cardiovasc Surg. (2012) 60:11–6. 10.1055/s-0031-1298068 22234488

